# Efficacy and safety analysis of dexamethasone + palonosetron in prevention of post-embolization syndrome after D-TACE: A retrospective study

**DOI:** 10.1097/MD.0000000000035433

**Published:** 2023-10-06

**Authors:** Haohao Lu, Chuansheng Zheng, Bin Liang, Xiangwen Xia

**Affiliations:** a Department of Radiology, Union Hospital, Tongji Medical College, Huazhong University of Science and Technology, Wuhan, China; b Hubei Province Key Laboratory of Molecular Imaging, Wuhan, China.

**Keywords:** complications, dexamethasone, drug-eluting beads, D-TACE, palonosetron, post-embolization syndrome, transcatheter arterial chemoembolization

## Abstract

To investigate the efficacy and safety of dexamethasone + palonosetron in the prevention of post-embolization syndrome after drug-eluting beads transcatheter arterial chemoembolization (D-TACE). The data of 278 patients who received D-TACE from January 2018 to December 2021 were collected and divided into 2 groups: D-TACE group (N = 145) and D-TACE + dexamethasone + palonosetron group (N = 133). The incidence of post-embolization syndrome and infection after D-TACE was assessed in both groups. Incidence of abdominal pain: D-TACE group versus D-TACE + dexamethasone + palonosetron group, 56.6% versus 40.6%, *P* = .008; incidence of fever: D-TACE group versus D-TACE + dexamethasone + palonosetron group, 40.0% versus 14.3%, *P* = .000; incidence of nausea: D-TACE group versus D-TACE + dexamethasone + palonosetron group, 61.4% versus 39.8%, *P* = .001; incidence of vomiting: D-TACE group versus D-TACE + dexamethasone + palonosetron group, 48.3% versus 21.1%, *P* = .000; incidence of infection: D-TACE group versus D-TACE + dexamethasone + palonosetron group, 1.4% versus 1.5%, *P* = .931. The combined use of dexamethasone and palonosetron before D-TACE can effectively reduce the incidence of post-embolization syndrome and reduce the degree of side effects, but it will not increase the risk of infection.

## 1. Introduction

Primary hepatocellular carcinoma is one of the malignant tumors with high incidence rate and mortality worldwide.^[1,2]^ In China, primary hepatocellular carcinoma (HCC) is the 4th most common malignant tumor and the second leading cause of cancer death, seriously threatening people’s lives and health.^[3]^ Since there are no specific symptoms and signs in the early stage of the disease, most patients have lost the opportunity of surgery when they are diagnosed. Transcatheter arterial chemoembolization (TACE) is one of the effective methods for the treatment of advanced hepatocellular carcinoma,^[4]^ which can significantly prolong the survival time of patients and benefit more and more patients with hepatocellular carcinoma.^[5,6]^ TACE is divided into conventional TACE and TACE with drug-eluting beads (D-TACE) according to the embolic agents used during the operation. Conventional transcatheter arterial chemoembolization refers to embolization with lipiodol emulsion. D-TACE is the use of drug-eluting microspheres loaded with chemotherapeutic drugs for tumor embolization, which has been widely used in clinic in recent years. The most common side effect after D-TACE is post-embolization syndrome,^[7,8]^ including abdominal pain, fever, nausea, and vomiting. Post-embolization syndrome will increase the physiological and psychological burden of patients, reduce their treatment compliance, prolong their hospitalization time, and increase their medical expenses.^[9]^ Since most patients with hepatocellular carcinoma in China have the background of cirrhosis, many patients are complicated with gastroesophageal varices. Severe post-embolization syndrome (especially nausea and vomiting) may lead to gastrointestinal bleeding after varices rupture, and even death. Therefore, the prevention and treatment of post-embolization syndrome is very important. The purpose of this study was to investigate the efficacy and safety of dexamethasone + palonosetron in the prevention of post-embolization syndrome after D-TACE.

## 2. Materials and methods

### 2.1. General information

Data of 278 patients treated with D-TACE in the interventional Department of Union Hospital Affiliated to Tongji Medical College of Huazhong University of science and technology from January 2018 to December 2021 were collected. Inclusion criteria: Primary hepatocellular carcinoma diagnosed clinically or pathologically, Barcelona clinic liver cancer (BCLC) stage A-C; Child-Pugh grade of liver function A to B, eastern cooperative oncology group 0 to 1; Age 18 to 70 years old; Molecular targeted drugs or immunotherapy were not used during the treatment.

#### 2.1.1. Exclusion criteria

1. Child-Pugh grade of liver function C, performance score eastern cooperative oncology group ≥ 2 points.
2. Severe coagulopathy and cannot be corrected.
3. Complete portal vein occlusion and less collateral vessels.
4. Combined with other vital organ dysfunction, such as heart, lung, kidney, and so on.
5. Allergic or contraindicated to the use of dexamethasone and palonosetron.

Patients were divided into 2 groups according to whether dexamethasone + palonosetron hydrochloride was used or not before D-TACE: D-TACE group (N = 145); D-TACE + dexamethasone + palonosetron group (N = 133). The collected data of patients included: gender, age, Child-Pugh classification of liver function, total bilirubin, albumin, alanine aminotransferase (ALT), aspartate aminotransferase (AST), etiology of liver cirrhosis, BCLC stage of tumor, history of previous vomiting, history of motion sickness, smoking history, history of drinking, preoperative fasting, and particle size of drug-eluting microspheres used during TACE.

### 2.2. Method

After disinfection and draping, the puncture site was locally anesthetized with 2% lidocaine, the femoral artery was punctured using the Seldinger technique, and a 5F vascular sheath was placed. A 5F Yashino catheter was used to cannulate the celiac trunk and superior mesenteric artery for angiography to identify the feeding artery of the tumor. Then a 2.7F microcatheter was superselectively cannulated into the tumor feeding artery, and an appropriate amount of drug-loaded microspheres were slowly injected for embolization, and the embolization endpoint was stagnation of forward blood flow in the tumor feeding artery. The particle size of the microspheres (CalliSpheres Drug-Loaded Embolic Microspheres, Hengrui Callisyn BioMedical Technology Co. Ltd., Suzhou, China, Registration No. FDA 510 (K): K173871) used in the operation was divided into 2 types: 100 to 300 um and 300 to 500 um, all loaded with 80 mg epirubicin. In the D-TACE + dexamethasone + palonosetron group, half an hour before TACE, dexamethasone 10 mg and palonosetron hydrochloride 0.5 mg were injected intravenously.

### 2.3. Outcome measures

Primary study endpoint: Incidence of abdominal pain, fever, nausea and vomiting 0 to 72 hours after D-TACE in both groups.

#### 2.3.1. Secondary study endpoints

1. Comparison of liver function 1 week after treatment between the 2 groups.
2. Comparison of the degree of abdominal pain within 1 week after treatment between the 2 groups (assessed by visual analogue scale [VAS] for pain).
3. Incidence of infection after D-TACE between the 2 groups (including positive blood culture or liver abscess).

### 2.4. Statistical methods

Statistical analysis was performed using SPSS software (Version 24.0, IBM, Armonk, New York). Number of cases (percentage) was used for enumeration data, and Chi-square test was used for differences, including Pearson Chi-Square and Fisher Exact Test. Measurement data were expressed as mean ± standard deviation, and 2 independent samples *t* test was used. *P* < .05 was considered to indicate a statistically significant difference.

## 3. Results

There was no statistically significant difference in gender, etiology of cirrhosis, history of vomiting, history of motion sickness, history of smoking, history of alcohol consumption, and preoperative fasting between the 2 groups using the Chi-square test (*P* > .05). The age of patients in the 2 groups was compared using the *t* test (*P* > .05), and there was no statistically significant difference. (shown in Table 1)

**Table 1:** Comparison of baseline data between the two groups.

			Group			
			D-TACE group (N = 145)	D-TACE + dexamethasone + palonosetron group (N = 133)	Chi-Square test (*P* value)	*t* test (*P* value)
Gender	Female	Count (%)	55 (37.9%)	41 (30.8%)	0.256	
	Male	Count (%)	90 (62.1%)	92 (69.2%)		
Etiology of cirrhosis	Hepatitis B	Count (%)	129 (89.0%)	120 (90.2%)	0.845	
	Hepatitis C	Count (%)	16 (11.0%)	13 (9.8%)		
History of vomiting	no	Count (%)	112 (77.2%)	98 (73.7%)	0.577	
	Yes	Count (%)	33 (22.8%)	35 (26.3%)		
History of motion sickness	No	Count (%)	108 (74.5%)	106 (79.7%)	0.321	
	Yes	Count (%)	37 (25.5%)	27 (20.3%)		
Smoking history	No	Count (%)	113 (77.9%)	108 (81.2%)	0.553	
	Yes	Count (%)	32 (22.1%)	25 (18.8%)		
Alcohol history	No	Count (%)	118 (81.4%)	110 (82.7%)	0.876	
	Yes	Count (%)	27 (18.6%)	23 (17.3%)		
Fasting before D-TACE	No	Count (%)	69 (47.6%)	72 (54.1%)	0.283	
	Yes	Count (%)	76 (52.4%)	61 (45.9%)		
Age (yr)	Mean ± SD		47.4 ± 11.6	48.5 ± 12.5		0.434

D-TACE = drug-eluting beads transcatheter arterial chemoembolization.

The treatment-related data between the 2 groups were compared (shown in Table 2), and there was no significant difference in liver function grade, BCLC stage, and particle size of microspheres used during operation (*P* > .05). The *t* test was used to compare the total bilirubin, albumin, ALT, and AST before treatment in the 2 groups (*P* > .05). One week after treatment, the comparison of total bilirubin, albumin, ALT and AST between the 2 groups showed no statistical difference (*P* > .05, shown in Table 3).

**Table 2:** Comparison of treatment-related data between the two groups.

			Group			
			D-TACE group (N = 145)	D-TACE + dexamethasone + palonosetron group (N = 133)	Chi-Square test (*P* value)	*t* test (*P* value)
posttreatment liver function	Child A	Count (%)	105 (72.4%)	90 (67.7%)	0.432	
	Child B	Count (%)	40 (27.6%)	43 (32.3%)		
Tumor BCLC staging	A	Count (%)	16 (11.0%)	14 (10.5%)	0.677	
	B	Count (%)	86 (59.3%)	73 (54.9%)		
	C	Count (%)	43 (29.7%)	46 (34.6%)		
Microsphere size in D-TACE (μm)	100–300	Count (%)	78 (53.8%)	64 (48.1%)	0.401	
	300–500	Count (%)	67 (46.2%)	69 (51.9%)		
pretreatment Bilirubin (μmol/L)	Mean ± SD		19.8 ± 6.3	17.6 ± 5.5		0.519
Pretreatment Albumin (g/L)	Mean ± SD		35.16 ± 7.42	34.25 ± 8.31		0.742
Pretreatment ALT (U/L)	Mean ± SD		41.6 ± 17.5	45.7 ± 18.9		0.162
Pretreatment AST (U/L)	Mean ± SD		40.5 ± 17.3	44.2 ± 17.7		0.276

ALT = alanine aminotransferase, AST = aspartate aminotransferase, BCLC = Barcelona clinic liver cancer, D-TACE = drug-eluting beads transcatheter arterial chemoembolization.

**Table 3:** Comparison of liver function one week after treatment between the two groups.

		Group		
		D-TACE group (N = 145)	D-TACE + dexamethasone + palonosetron group (N = 133)	*t* test (*P* value)
posttreatment Bilirubin (μmol/L)	Mean ± SD	23.6 ± 5.8	21.3 ± 7.1	.230
posttreatment Albumin (g/L)	Mean ± SD	32.45 ± 8.06	31.60 ± 6.92	.481
posttreatment ALT (U/L)	Mean ± SD	78.5 ± 26.1	74.0 ± 23.5	.332
posttreatment AST (U/L)	Mean ± SD	69.3 ± 19.7	62.8 ± 12.4	.184

ALT = alanine aminotransferase, AST = aspartate aminotransferase, D-TACE = drug-eluting beads transcatheter arterial chemoembolization.

The incidence rate of post-embolization syndrome after D-TACE was compared between the 2 groups (shown in Table 4). The incidence of abdominal pain (56.6% vs 40.6%), fever (40.0% vs 14.3%), nausea (61.4% vs 39.8%), and vomiting (48.3% vs 21.1%) was statistically different between the 2 groups with *P* < .05. The incidence rate of infection after D-TACE was also compared between the 2 groups (shown in Table 4). There was no statistical difference between the 2 groups (*P* > .05).

**Table 4:** Comparison of incidence rate of post-embolization syndrome and infection after D-TACE between the two groups.

			Group		Chi-Square test (*P* value)
			D-TACE group (N = 145)	D-TACE + dexamethasone + palonosetron group (N = 133)	
Abdominal pain	No	Count (%)	63 (43.4%)	79 (59.4%)	.008
	Yes	Count (%)	82 (56.6%)	54 (40.6%)	
Fever	No	Count (%)	87 (60.0%)	114 (85.7%)	.000
	Yes	Count (%)	58 (40.0%)	19 (14.3%)	
Nausea	No	Count (%)	56 (38.6%)	80 (60.2%)	.001
	Yes	Count (%)	89 (61.4%)	53 (39.8%)	
Vomiting	No	Count (%)	75 (51.7%)	105 (78.9%)	.000
	Yes	Count (%)	70 (48.3%)	28 (21.1%)	
Infection	No	Count (%)	143 (98.6%)	131 (98.5%)	.931
	Yes	Count (%)	2 (1.4%)	2 (1.5%)	

D-TACE = drug-eluting beads transcatheter arterial chemoembolization.

The VAS score of abdominal pain within 1 week after treatment was compared between the 2 groups (shown in Table 5). On days 1 to 2 after treatment, the abdominal pain VAS score in the D-TACE + dexamethasone + palonosetron group was lower than that in the D-TACE group (*P* < .05). On the 3rd to 7th day after treatment, the VAS scores of abdominal pain in the 2 groups were gradually decreased, with *P* value > .05, indicating no statistical difference.

**Table 5:** Comparison of VAS score of abdominal pain within one week after treatment between the two groups.

	Group		*t* test (*P* value)
	D-TACE group (N = 145)	D-TACE + dexamethasone + palonosetron group (N = 133)	
Day 1	7.9 ± 2.8	5.7 ± 2.4	.025
Day 2	6.7 ± 3.3	4.2 ± 2.6	.031
Day 3	5.3 ± 3.1	4.4 ± 2.5	.104
Day 4	4.6 ± 2.7	4.1 ± 3.2	.387
Day 5	3.9 ± 1.8	3.7 ± 2.2	.592
Day 6	3.6 ± 2.0	3.3 ± 1.9	.558
Day 7	3.2 ± 1.1	2.9 ± 0.8	.745

D-TACE = drug-eluting beads transcatheter arterial chemoembolization, VAS = visual analogue scale.

## 4. Discussion

TACE is one of the effective treatments for unresectable HCC. Its main principle is that after artery puncture to establish access, the catheter is super selective intubated into the blood supply artery of the tumor, and chemotherapeutic drugs and embolic agents are injected.^[10]^ On the 1 hand, chemotherapeutic drugs can induce tumor cell apoptosis and inhibit tumor cell proliferation; On the other hand, tumor blood supply artery embolization leads to tumor cell ischemia, hypoxia and necrosis. Due to the slow-release effect of drug-loaded microspheres, D-TACE can maintain a high blood drug concentration in the local tumor for a long time, which is reliable in the treatment of hepatocellular carcinoma and plays a very important role in the treatment of hepatocellular carcinoma.^[11]^ The most common side effect after D-TACE is post-embolization syndrome, including pain, fever, nausea and vomiting.^[12]^ The possible causes of post-embolization syndrome after D-TACE are^[13,14]^: Chemotherapy drugs used during operation stimulate chemoreceptors in the gastrointestinal tract through blood circulation, resulting in nausea, and vomiting; Tissue ischemia and hypoxia after TACE lead to pain; Visceral tissue (especially liver and gall) ischemia and pain can also lead to nausea and vomiting; Ischemia, hypoxia and necrosis of HCC tissue lead to aseptic inflammation, induce the release of a variety of inflammatory transmitters, and lead to fever, nausea and vomiting; Trauma and stress in D-TACE will lead to the release of dopamine, adrenaline and other transmitters, resulting in pain, nausea, and vomiting; Patient factors, such as young women, no smoking history, previous history of postoperative vomiting, history of motion sickness, etc. Severe post-embolization syndrome will increase patients pain and lead to increased psychological burden. So as to make the patients fear the treatment and reduce the treatment compliance. At the same time, severe post-embolization syndrome will increase the risk of perioperative period, prolong the hospitalization time, increase the medical expenses of patients, and even lead to the death of patients.^[15]^

Dexamethasone, as a steroid hormone, has the effects of anti-inflammatory, anti endotoxin, anti immune, anti shock, and enhancing stress response. It is widely used in the treatment of a variety of diseases. Dexamethasone takes glucocorticoid receptor as its target. It plays a very important role in inflammation and immune response by inducing apoptosis of immune cells and inhibiting the release of inflammatory transmitters. The occurrence of post-embolization syndrome after D-TACE is partly due to the aseptic inflammation caused by tumor tissue ischemia and hypoxia necrosis and the release of inflammatory mediators. Dexamethasone can effectively inhibit the inflammatory response and reduce the manifestations of inflammation (such as fever and pain). Many studies have shown that,^[16]^ dexamethasone plays a very effective role in preventing postoperative nausea and vomiting (PONV) and chemotherapy-induced nausea and vomiting (CINV). D-TACE is also a minimally invasive operation. The occurrence of nausea and vomiting after D-TACE is similar to PONV. Jieting Liu et al^[17]^ reported that dexamethasone can effectively reduce the incidence of PONV with few complications. Hamed Moheimani et al^[18]^ found that a single preoperative use of dexamethasone can also effectively reduce the occurrence of PONV. Chemotherapeutic drugs are also used in D-TACE. These chemotherapeutic drugs have a medium to high-risk of emesis. The occurrence of nausea and vomiting after D-TACE is similar to CINV. Dexamethasone also plays a very important role in the prevention and treatment of CINV.^[19]^ Some studies have shown that dexamethasone can relieve postoperative pain and prolong the duration of postoperative analgesia. Tenghui Zhang et al^[20]^ found that a single dose of 8mg dexamethasone can effectively reduce postoperative pain and shorten hospital stay. Leilai Xu et al^[21]^ reported that dexamethasone can not only reduce the incidence of PONV, but also reduce postoperative pain. It is reported by Elizabeth A Klag et al^[22]^ that dexamethasone can effectively control the occurrence of postoperative pain, nausea and vomiting.

Palonosetron hydrochloride is a second generation 5-Hydroxytryptamine 3 (5-HT3) receptor antagonist, which can play an antiemetic role in the central and gastrointestinal tract.^[23]^ Compared with the first generation 5-HT3 receptor antagonist, palonosetron inhibits the 5-HT3 receptor through competitive inhibition and allosteric inhibition, and its affinity with the receptor is higher. The affinity between palonosetron and 5-HT3 receptor is nearly 100 times that of ondansetron and granisetron, which can effectively control the occurrence of nausea and vomiting.^[24]^ The half-life of palonosetron hydrochloride was nearly 40 hours, which was 4 to 10 times that of dorasetron, granisetron or ondansetron. It was found that palonosetron was more effective in the delayed period than the first generation 5-HT3 receptor antagonist.^[25]^ In addition, it has been found in a number of phase II to III clinical trials that palonosetron hydrochloride has fewer adverse reactions and higher safety. Morganroth Joel et al^[26]^ found that palonosetron had no effect on cardiac repolarization measured by corrected QT interval even at the super therapeutic dose. Because palonosetron hydrochloride has many advantages, more and more doctors apply it to the prevention of PONV and CINV. Rohit Balyan et al^[27]^ reported that among high-risk patients undergoing gynecological laparoscopic surgery, palonosetron had better anti nausea effect 2 to 24 hours after operation than ondansetron, less demand for remedial antiemetic drugs, lower incidence of total PONV, and the effect of 0 to 2 hours and 24 to 48 hours after operation was equivalent to ondansetron. It is reported by Preet Mohinder Singh et al^[28]^ that palonosetron is as safe and more effective as placebo, ramosetron, granisetron, and ondansetron in the prevention of delayed PONV. For early PONV, the efficacy was higher than that of placebo, granisetron, and ondansetron. It is found by Tanvi Bhargava et al^[29]^ that there was significant difference in incidence of PONV between palonosetron group and ondansetron group at 6 hours (12.5% vs 32.1%, *P* = .013) and 72 hours (1.8% vs 33.9%, *P* < .001) in live-related renal transplant recipients. Mohd Atesham Khan et al^[30]^ reported that for female patients undergoing laparoscopic cholecystectomy, intravenous administration of palonosetron (0.075 mg) is as effective as dexamethasone (4 mg) in preventing vomiting without any adverse effects. It is reported by Weihua Tian et al^[31]^ that palonosetron is well tolerated in the Chinese population and can effectively prevent nausea and vomiting caused by acute and delayed chemotherapy. Yunpeng Yang et al^[32]^ found that compared with tropisetron, palonosetron is a highly selective antiemetic drug and an effective treatment for CINV control in tumor patients. It is found by Tatsuhiko Sakamoto et al^[33]^ that the administration of palonosetron and dexamethasone before TACE can significantly prevent the incidence and reduce the severity of gastrointestinal symptoms, especially in the acute phase.

Based on the therapeutic effects of dexamethasone and palonosetron hydrochloride, in this study, patients in the D-TACE + dexamethasone + palonosetron group were injected with dexamethasone 10 mg + palonosetron 0.5 mg intravenously before D-TACE. Our results showed that the incidence of abdominal pain: D-TACE group versus D-TACE + dexamethasone + palonosetron group, 56.6% versus 40.6%, *P* = .008; The incidence of fever: D-TACE group versus D-TACE + dexamethasone + palonosetron group, 40.0% versus 14.3%, *P* = .000; The incidence of nausea: D-TACE group versus D-TACE + dexamethasone + palonosetron group, 61.4% versus 39.8%, *P* = .001; The incidence of vomiting: D-TACE group versus D-TACE + dexamethasone + palonosetron group, 48.3% versus 21.1%, *P* = .000. Dexamethasone plus palonosetron before D-TACE can effectively reduce the occurrence of post-embolization syndrome, and the degree of abdominal pain 1 week after D-TACE is significantly reduced. Vito Lorusso et al^[34]^ reported that single dose palonosetron and dexamethasone can prevent CINV in most patients. It is found by Luigi celio et al^[35]^ that palonosetron plus 1-day dexamethasone provides an effective treatment option for the prevention of CINV. It is reported by Eunah Cho et al^[36]^ that the incidence of PONV in palonosetron + dexamethasone group was significantly lower than that in palonosetron group (61.5% vs 77.1%; *P* = .019) 0 to 48 hours after operation. Compared with the palonosetron group, the severity of nausea at 0 to 6 hours after operation in the palonosetron + dexamethasone group was significantly lower (no/mild/moderate/severe: 49/22/15/10 vs 36/16/25/19, *P* = .008). Amit Kumar et al^[37]^ reported palonosetron with dexamethasone is more effective than ondasetron with dexamethasone for prevention of PONV in post-chemotherapy ovarian cancer surgeries receiving opioid-based patient-controlled analgesia. Anish n g Sharma et al^[38]^ found that among patients undergoing elective laparoscopic hysterectomy under general anesthesia, palonosetron combined with dexamethasone is more effective in the treatment of early, delayed and long-term PONV than ondansetron combined with dexamethasone.

Because dexamethasone can inhibit the immune function of the body, it may induce or aggravate infection in use. Especially for tumor patients, the resistance of the body decreases, and the risk of infection is higher. However, existing studies have shown that,^[39]^ the use of dexamethasone in TACE does not increase the incidence of infection after TACE, and the use of dexamethasone in TACE is safe. The results of this study showed that the incidence of infection: D-TACE group versus D-TACE + dexamethasone + palonosetron group, 1.4% versus 1.5%, *P* = .931, there was no statistical difference, which was consistent with the results of other studies. It is reported by Sadahisa Ogasawara et al^[40]^ that dexamethasone is more effective and safer in preventing post-embolization syndrome after TACE than the control group. It is foung by Haohao Lu et al^[41]^ that the lipiodol + dexamethasone emulsion can significantly reduce the incidence rate of post-embolization syndrome after TACE, with exact effect and high safety, and it did not increase the incidence of infection after TACE.

## 5. Conclusion

Post-embolization syndrome after D-TACE is one of the common side effects. Its causes may include operation trauma, ischemia and hypoxia of tumor tissue, aseptic inflammation, intraoperative use of chemotherapy drugs, ischemia of liver and bile duct, intraoperative stress of TACE, patient factors, and absorption of necrotic substances after embolization. The combination of dexamethasone and palonosetron before D-TACE can effectively reduce the incidence of post-embolization syndrome and the degree of side effects, but will not increase the risk of infection.

The inadequacy of this study is that the data comes from a single center, and it is a retrospective study with limited sample size. In the later stage, multi center, large sample and prospective research are feasible to provide more help for clinical work.

## Acknowledgements

Thank Pro. Fan Yang for her help in English writing. Thank Dr Xin Li for his help in data collection. Thank Dr Hongjie Fan for his help in reference. Thank you for all those who offer help and support in this research. They have also confirmed that their names can be disclosed.

## Author contributions

**Conceptualization:** Haohao Lu, Chuansheng Zheng.

**Data curation:** Chuansheng Zheng, Bin Liang.

**Formal analysis:** Chuansheng Zheng, Bin Liang, Xiangwen Xia.

**Investigation:** Haohao Lu, Xiangwen Xia.

**Methodology:** Haohao Lu, Chuansheng Zheng, Xiangwen Xia.

**Writing – original draft:** Haohao Lu.

**Writing – review & editing:** Chuansheng Zheng, Bin Liang.
